# Menstruation angina: a case report

**DOI:** 10.1186/1752-1947-3-6618

**Published:** 2009-03-27

**Authors:** Wai Kah Choo

**Affiliations:** 1 Cardiology Department, Whipps Cross University Hospital, London E11 1NR, UK

## Abstract

**Introduction:**

Menstruation is commonly associated with migraine and irritable bowel but is rarely correlated with angina or myocardial ischaemia. Only a small number of cases have been reported suggesting a link between menstruation and myocardial ischaemic events.

**Case presentation:**

A case of menstruation angina is reported in order to raise awareness of this association. A 47-year-old South Asian woman presented with recurrent chest pains in a monthly fashion coinciding with her menstruations. Each presentation was associated with troponin elevation. Angioplasty failed to resolve her symptoms but she eventually responded to hormonal therapy.

**Conclusions:**

The possibility of menstruation angina should always be taken into account in any female patients from puberty to menopause presenting with recurrent chest pains. This can allow an earlier introduction of hormonal therapy to arrest further myocardial damage.

## Introduction

While some live through menstrual cycles as part and parcel of their lives, the cyclical fluctuation of sex hormones disturbs the lives of many others. To date, menstruation has established effects on migraine, epilepsy, asthma, irritable bowel syndrome and diabetes [1].

The few suffering from 'menstruation angina' or 'catamenial angina' may not have earned the attention they deserve. Research into the relationship between coronary events and menstrual cycles is still in its infancy, but preliminary trials have so far shown compelling evidence.

## Case presentation

A 47-year-old South Asian woman presented with a 3-month history of recurrent left-sided chest pains. Initially, her pains were non-exertional and were relieved by massages and antacids. The onset of her pains usually coincided with the second or third day of her menstruation each month and usually lasted up to 4 days. Rests frequently eased her pains, hence she did not seek medical attention.

She eventually presented to our Emergency Department in the fourth month. She had sudden onset sharp chest pain, 10/10 in intensity, which radiated up to her jaw and travelled down to her arms. The pain was non-exertional and sublingual nitrates did not help. She also complained of diaphoresis, hot flushes and persistent lethargy. This presentation coincided with the second day of her menstruation.

Aside from a raised Body Mass Index (BMI) of 37.2 and anaemia, she did not have any other cardiac risk factors. Her fasting blood glucose was 6.8 and she did not have a history of ischaemic heart disease. These risk factors had not changed over the last year.

Her menstrual periods were usually regular in interval and she rarely suffered from menorrhagia. She had had two myomectomies about 25 years ago. A fibroid of the size of a 3-month-old foetus was discovered in a recent laparotomy. She had three failed attempts of in-vitro fertilization (IVF). There was no family history of ischaemic heart disease or gynaecological conditions. She did not take any medication on a regular basis.

On examination, she was found to have peripheral and central cyanosis. There were no other relevant findings. Her electrocardiogram showed ST-segment elevation and Q waves in the anterior leads and ST-segment depression in the inferior leads. This together with a rise in troponin T and creatine kinase levels to 50ng/ml and 2045U/L, respectively was suggestive of a ST segment elevation myocardial infarction (STEMI). Other abnormalities in the serum laboratory studies were a raised C-reactive protein (CRP) of 70mg/L and haemoglobin of 9.2g/dL. She was transfused with a unit of blood as a result. A coronary angiogram revealed a left anterior descending (LAD) artery occlusion. A stent was inserted.

Her chest pain recurred the following month, again coinciding with her menstruation. The nature of her pain in this admission was similar to that in her first admission although it was reported to be milder in severity. Her electrocardiogram this time showed ischaemic changes only to the inferior region. Troponin T was elevated to 3.2ng/mL. Oestrogen and progesterone levels were consistently normal to her phases in menstruation.

Given that the troponin level usually takes about 10 days to decline to normality post-myocardial infarction, troponin detected in this second admission may be a misnomer and may be attributed to the slow decline in troponin from her first admission. In that case, the diagnosis in this admission would be unstable angina rather than STEMI, as per definition [2].

Although the failure of symptom resolution after angioplasty may indicate an early stent failure or incomplete coronary revascularization, it could also indicate the presence of another underlying pathology. Dynamic obstructions due to intense focal spasm of arteries as seen in Prinzmetal's angina could also explain the persistence of her symptoms. Coronary vasospasm itself may also trigger a rise in troponin [3]. It was agreed at that time that she should be managed conservatively but a coronary angiogram should be repeated if her symptoms changed in nature, character or frequency.

Monthly serial electrocardiograms were done. Ergonovine or acetylcholine could be used to induce coronary vasospasm during coronary angiography but was not attempted due to safety concerns and lack of prognostic values. A trial of luteinizing hormone releasing hormone (LHRH) analogues in an attempt to induce early menopause only brought limited benefit although she experienced multiple relapses. While LHRH might be a choice to halt menstruation, it does not stop the underlying cyclical oestrogen decline. Instead, it completely removes oestrogen.

Ultimately, hormonal replacement therapy (oestrogen replacement) was used to address the cyclical oestrogen decline with good response. Her symptoms largely resolved in the subsequent 12 months, with only one episode of repeat chest pain. An angiography performed eventually showed patent arteries and stent. She was planned to continue with antithrombotic modulation (aspirin and clopidogrel) for her underlying atherosclerotic disease.

## Discussion

Small guanosine triphosphate (GTP)-binding protein Rho and its effector Rho-kinase play an important role in smooth muscle contraction and actin cytoskeleton organization [4]. Porcine and human artery experiments *in vivo* have demonstrated that Rho-kinase can induce inflammatory atherosclerotic lesions and trigger coronary vasospastic responses through inhibition of myosin light chain phosphate [5]. On the other hand, long-term inhibition of Rho-kinase can resolve coronary vasospastic activities [6].

At supraphysiological concentrations, oestrogen is known to inhibit Rho-kinase mRNA expression in human coronary vascular smooth muscle cells (VSMC) averting vasospasm [7]. Acting through two receptors, α and β, oestrogen is also known to inhibit the influx of extracellular calcium into VSMC and to up-regulate genes governing vasodilatory enzymes (that is to say, prostacyclin synthase and nitric oxide synthase) [8]. These mechanisms will reduce vascular tone in the long term.

At physiological concentrations, oestrogen was shown to cause rapid release of nitric oxide without any prerequisite genetic alterations [9]. This, together with the cGMP-mediated pathways, will open the calcium-activated potassium channels relaxing smooth muscles [8].

Oestrogen α-receptors are thought to be more important for nitric oxide synthase activity [10]. The variability in number of α- and β-oestrogen receptors from person to person may explain why some women are more prone to heart disease than others.

**Figure 1:**
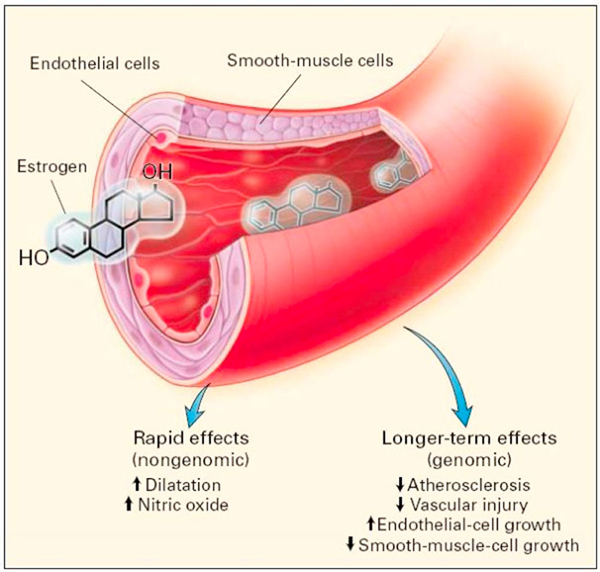
**Direct effects of oestrogen on blood vessels**.

The effects of oestrogen on blood vessels can be simplified into rapid (non-genomic) effects and long-term (genomic) effects as illustrated by Mendelsohn and Karas in Figure 1[10]. Other long-term cardioprotective effects of oestrogen including the acceleration in endothelial cell regeneration post-vascular injury and the reduction in atherosclerosis may be less relevant to the pathogenesis of menstruation angina [11].

The physiological fluctuation in oestrogen and progesterone levels occurs in a 28-day cycle in a pre-menopausal woman. Oestrogen level is lowest in the menstrual phase (Day 1-5) followed by a gradual rise peaking around Day 14 when ovulation occurs. Thereafter, oestrogen level tails off making way for the next cycle.

It is known that post-menopausal women suffer from an increased risk of atherosclerotic and vasospastic disorders after losing oestrogen's cardioprotective effects [6]. The reduction in oestrogen level at the commencement of each menstrual cycle in a premenopausal woman may mimic the lack of oestrogen post-menopausal women encounter. This coincides with a higher risk of coronary events, as seen in our patient. In a study by Lloyd *et al.*, all 10 young female participants developed 1-mm ST-segment depression and chest pain quicker when their oestrogen levels were lowest [12].

Clark *et al.* observed electrocardiogram changes in 12 healthy female volunteers in three phases: the menstruation (days 2-3), pre-ovulation (days 12-13) and post-ovulation (day 19) phases [13]. Six of the volunteers developed ST-segment depression in stress tests during the menstruation and pre-ovulation phases or both, when oestrogen levels were lowest. When progesterone levels were highest at day 19, there were no greater electrocardiographic abnormalities.

Kawano *et al.* examined the effects of oestrogen on arterial function and ischaemic symptoms in 10 women [14]. The diameter and flow velocity in the brachial arteries were presumed to reflect those of coronary arteries since they have related endothelial functions [14]. Vasodilatation was found to be greatest at mid-cycle when oestrogen levels were high. When oestrogen levels were lowest in the menstrual phase, the patients had more ischaemic events with corresponding ST segment changes.

Failing all, vasospastic angina can be treated in isolation. High doses of calcium antagonists and anti-alpha adrenergic drugs, such as guanethidine or clonidine, can suppress persistent attacks [15]. The use of anti-oxidant vitamins C and E may also improve endothelial function and decrease vascular reactivity in vasospastic angina [15]. Rho-kinase inhibitor, still in its experimental stages, is aimed at preventing VSMC-induced vasospasm [15].

Coronary bypass surgery may be indicated in patients with obstructive coronary artery disease in addition to coronary vasospasm as in this scenario. However, in our patient, it may not be immediately necessary given that she has a patent stent.

## Conclusion

Laboratory and genetic studies have confirmed the cardioprotective effects of oestrogen. To date, we only have three notable trials to illustrate that angina can be set off by diminished oestrogen levels during menstruation, one of them accomplished almost two decades ago.

In years to come, more studies need to be conducted. A substantial number of patients may be suffering from this condition without our knowledge. Episodic chest pains if mild may be overshadowed by more common symptoms of menstruation as mentioned. Not until then may guidelines be drawn to change our clinical practice in women's health.

We should also bear in mind that, although episodic chest pains in women may be attributed to cyclical oestrogen fluctuations, atherosclerotic coronary artery disease is still more common. Therefore, coronary angiography should not be delayed in any case.

## Abbreviations

BMI: body mass index; cGMP: cyclic guanosine monophosphate; CRP: C-reactive protein; GTP: guanosine triphosphate; IVF: in-vitro fertilization; LAD: left anterior descending; LHRH: luteinizing hormone releasing hormone; mRNA: messenger ribonucleic acid; STEMI: ST segment elevation myocardial infarction; VSMC: vascular smooth muscle cells.

## Consent

Written informed consent was obtained from the patient for publication of this case report. A copy of the written consent is available for review by the Editor-in-Chief of this journal. The patient does not wish to have her angiogram images published.

## Competing interests

The author declares that he has no competing interests.

## Authors' contributions

WKC was involved in the progress and treatment of this patient and in the writing of this case.
